# Retraction Note: Role of dopamine D_2_ receptors in ischemia/reperfusion induced apoptosis of cultured neonatal rat cardiomyocytes

**DOI:** 10.1186/s12929-025-01184-0

**Published:** 2025-09-12

**Authors:** Hong-zhu Li, Jin Guo, Jun Gao, Li-ping Han, Chun-ming Jiang, Hong-xia Li, Shu-zhi Bai, Wei-hua Zhang, Guang-wei Li, Li-na Wang, Hong Li, Ya-jun Zhao, Yan Lin, Ye Tian, Guang-dong Yang, Rui Wang, Ling-yun Wu, Bao-feng Yang, Chang-qing Xu

**Affiliations:** 1 Department of Pathophysiology, Harbin Medical University, Harbin, People’s Republic of China; 2 Department of Child Cerebral Palsy, The Third Clinical Hospital of Jiamusi University, Jiamusi, People’s Republic of China; 3 The First Hospital, Harbin, People’s Republic of China; 4 Department of Physiology, Wenzhou Medical College, Wenzhou, People’s Republic of China; 5 Department of Pathophysiology, Qiqihar Medical University, Qiqihar, People’s Republic of China; 6 Department of Biology, Lakehead University, Thunder Bay, ON P7B5E1 Canada; 7 Department of Pharmacology, University of Saskatchewan, Saskatoon, SK S7H 5E5 Canada; 8 Department of Pharmacology, Harbin Medical University, Harbin, People’s Republic of China; 9 Bio-Pharmaceutical Key Laboratory of Heilongjiang Province, Harbin, People’s Republic of China

**Retraction Note: Journal of Biomedical Science (2011) 18:18** 10.1186/1423-0127-18-18

The Editor-in-Chief has retracted this article. After publication, concerns were raised regarding some of the data presented in the figures, specifically:

- Fig. 1B I/R and Bromocriptine images appear to share some overlapping features;
- Fig. 1B Bromocriptine image appears to show a duplicated cell on the right side of the image (top and bottom);
- Fig. 5A actin lanes 3 and 4 appear highly similar to Fig. 5C actin lanes 1 and 2;
- Fig. 5B actin blot appears highly similar to Fig. 6A, B and C actin;
- Fig. 5B, 6A, B and C actin blots, lanes 3 and 4 appear highly similar;
- Fig. 6A Fas, 6B Fas-L and 6C Caspase-3 blots appear to have inconsistencies in the blot backgrounds.

The Editor-in-Chief therefore no longer has confidence in the presented data.

Hong-zhu Li and Jun Gao do not agree with this retraction. None of the other authors have responded to any correspondence from the editor or publisher about this retraction.

